# 159 Qualitative insights into the skills development, health outcomes, and school experience of adolescent peer-leaders of a physical activity programme in second level schools

**DOI:** 10.1093/eurpub/ckae114.183

**Published:** 2024-09-26

**Authors:** Kathleen McNally, Elaine Murtagh, Kwok Ng, Caera Grady, Catherine Woods

**Affiliations:** Physical Activity for Health Research Centre, Health Research Institute, Department of Physical Education and Sports Sciences, University of Limerick, Ireland; Physical Activity for Health Research Centre, Health Research Institute, Department of Physical Education and Sports Sciences, University of Limerick, Ireland; Physical Activity for Health Research Centre, Health Research Institute, Department of Physical Education and Sports Sciences, University of Limerick, Ireland; Faculty of Education, University of Turku, Rauma, Finland; Institute of Innovation and Sports Science, Lithuanian Sports University, Kaunas, Lithuania; Physical Activity for Health Research Centre, Health Research Institute, Department of Physical Education and Sports Sciences, University of Limerick, Ireland; Physical Activity for Health Research Centre, Health Research Institute, Department of Physical Education and Sports Sciences, University of Limerick, Ireland

## Abstract

**Purpose:**

The Active School Flag (ASF) is a whole-of-school, physical activity (PA) programme in Ireland. At second level, student leadership is central to ASF, as adolescents aged 15–16 years in Transition Year (TY), are peer-leaders (PL) of ASF in their school. TY is a one-year programme unique to Ireland, that provides adolescents with skills and personal development opportunities. PLs receive leadership training and have a dedicated weekly class to plan the delivery of whole-of-school PA events and surveys. This study explored the impact on PL skills development, health outcomes, and school experience.

**Methods:**

In April and May 2023, focus groups were conducted with PL (n = 33, 67% female) in eight schools during their second year of ASF. PL were asked about their experience of implementing ASF.

Data were analysed using reflexive thematic analysis informed by Braun and Clarke, (2019). Five themes and 29 subthemes were generated.

**Results:**

Themes included: (1) Enhanced personal development through ASF, (2) ASF builds community in the school, (3) Value and recognition of ASF in the school community, (4) Without ASF school would be plainer, (5) PL experience of their participation in ASF.

Findings demonstrate the impact of key ASF intervention components on PL skills, health, and school experience. PL developed transferable skills including verbal and written communication (theme 1) through dissemination of whole-of-school survey results and sharing PA messages with school members. Shared leadership training supported PL to feel part of a group (theme 2) and facilitated a comfortable social and learning environment (theme 4). Attending ASF weekly timetabled class stimulated a positive mood among PL (theme 5). Although PL felt school members supported them (theme 3) during whole-of-school PA events, some PL experienced anxiety and frustration working with school members (theme 5).

**Conclusions:**

Results provide valuable information to inform school staff and management how leading ASF can influence adolescents, including developing transferable skills, enhancing social and mental health, and supporting a positive school experience.

These insights highlight the potential impact of a whole-school PA programme on a broad range of outcomes, demonstrating the added value of student-led initiatives.

**Funding Source:**

Mayo Education Centre, Healthy Ireland

